# Does Cholinergic Stimulation Affect the P2X7 Receptor-Mediated Dye Uptake in Mast Cells and Macrophages?

**DOI:** 10.3389/fncel.2020.548376

**Published:** 2020-10-28

**Authors:** Dilyara Nurkhametova, Andrei Siniavin, Maria Streltsova, Denis Kudryavtsev, Igor Kudryavtsev, Raisa Giniatullina, Victor Tsetlin, Tarja Malm, Rashid Giniatullin

**Affiliations:** ^1^A.I. Virtanen Institute for Molecular Sciences, University of Eastern Finland, Kuopio, Finland; ^2^Laboratory of Neurobiology, Kazan Federal University, Kazan, Russia; ^3^Department of Molecular Neuroimmune Signalling, Shemyakin-Ovchinnikov Institute of Bioorganic Chemistry, Russian Academy of Sciences, Moscow, Russia; ^4^Department of Immunology, Shemyakin-Ovchinnikov Institute of Bioorganic Chemistry, Russian Academy of Sciences, Moscow, Russia; ^5^Department of Immunology, Institute of Experimental Medicine, St. Petersburg, Russia; ^6^Department of Fundamental Medicine, Far Eastern Federal University, Vladivostok, Russia

**Keywords:** acetylcholine, nicotin, neuroinflammation, neurodegeneration, P2X7, mast cell, cholinergic anti-infl ammatory pathway, macrophage

## Abstract

**Background**: Extracellular ATP is a powerful trigger of neuroinflammation by activating immune cells *via* P2X7 receptors. Acetylcholine and nicotinic agonists inhibit ATP-triggered proinflammatory cytokines *via* the so-called “cholinergic anti-inflammatory pathway” (CAP). However, it remains unclear as to what stage of ATP-induced signaling cholinergic agents provide this anti-inflammatory effect. Using the specific property of P2X7 receptor to open a pathway permeable to large molecules, associated with activation of inflammasome, we studied the action of cholinergic agents on this key event in CAP activation.

**Methods**: Freshly isolated mouse peritoneal mast cells and primary human macrophages were used. To assess P2X7 channel opening, the permeability to the fluorescent dye YO-PRO1 or ethidium bromide (EtBr) was measured by flow cytometry. Expression of nicotinic receptors was probed in macrophages with the fluorescently labeled α-bungarotoxin or with patch-clamp recordings.

**Results**: ATP opened P2X7 ion channels in mast cells and macrophages permeable to YO-PRO1 or EtBr, respectively. This stimulatory effect in mast cells was inhibited by the specific P2X7 antagonist A839977 confirming that YO-PRO1 uptake was mediated *via* ATP-gated P2X7 ion channels. Cholinergic agents also slightly induced dye uptake to mast cells but not in macrophages, which expressed functional α7 nicotinic receptors. However, both in mast cells and in macrophages, acetylcholine and nicotine failed to inhibit the stimulatory effect of ATP on dye uptake.

**Conclusion**: These data suggest that in immune cells, cholinergic agents do not act on P2X7 receptor-coupled large pore formation but can mediate the anti-inflammatory effect underlying CAP downstream of ATP-driven signaling.

## Introduction

In distinct neurological disorders ranging from migraine (Yücel et al., 2016; Becher et al., 2017; Khaiboullina et al., 2017; Degan et al., 2018) to neurodegeneration (Griffin et al., 1998; Ng et al., 2018), much attention was attracted to the role of the harmful neuroinflammation associated with elevated levels of proinflammatory cytokines of the interleukin-1 (IL-1) family. Production and release of IL-1β and related cytokines can be activated *via* purinergic ATP-gated P2X7 receptors (Ferrari et al., 2006; Rathinam et al., 2012; Di Virgilio et al., 2017; Wang et al., 2020), which are expressed in apparently all immune cells (Jacob et al., 2013; Takenaka et al., 2016).

In mice lacking P2X7 receptors, ATP does not lead to release of IL-1β from LPS-primed cells (Solle et al., 2001). Consistent with this finding, the research groups are searching for the novel efficient P2X7 antagonists to counteract the excessive neuroinflammation (Gonzaga et al., 2019). The prominent property of the P2X7 receptor subtype is the opening of the large pore permeable to cationic dyes such as YO-PRO1 and ethidium bromide (EtBr; North, 2002). It is a debatable issue whether this pore is located within the P2X7 receptor or in associated membrane proteins such as pannexins (Pelegrin and Surprenant, 2006; Kanellopoulos and Delarasse, 2019). In any case, opening of this pore is associated with large K^+^ efflux, which is one of the signals activating the NLRP3 inflammasome involved in the secretion of IL-1β from human macrophages (Gicquel et al., 2015; Kanellopoulos and Delarasse, 2019). NLRP3 inflammasome activation typically requires priming with agents like the TLR4 agonist LPS (Gicquel et al., 2014; Parzych et al., 2017) and depends on membrane composition (Di Virgilio et al., 2018). P2X7 receptor-induced NLRP3 inflammasome activation has been linked to common neurological disorders such as Alzheimer’s disease (Thawkar and Kaur, 2019). Interestingly, activation of P2X7 receptors, apart from ATP, can be induced by noncanonical agonists including the amyloidogenic β peptide and serum amyloid A (Di Virgilio et al., 2018).

The cholinergic pathway, activated by acetylcholine or its analogs, has been proposed to dampen down inflammatory processes in many neurological diseases (Borovikova et al., 2000; Wang et al., 2003; Rosas-Ballina and Tracey, 2009). This type of the neuromodulation, termed as the *cholinergic anti-inflammatory pathway* (CAP; Borovikova et al., 2000), has attracted much attention as a promising approach to limit excessive inflammation. Monocytes and closely related macrophages are the classical models to study inflammasome activation, secretion of proinflammatory cytokines, and CAP mechanisms. Several research groups have demonstrated that cholinergic agonists, including acetylcholine and nicotine, operating *via* α7, α9, or α10 receptor subtypes, inhibit ATP-induced IL-1β release from immune cells, including human and rat monocytes (Báez-Pagán et al., 2015; Hecker et al., 2015; Zakrzewicz et al., 2017; Hiller et al., 2018). We and others have shown that stimulation of α7 receptors decreases the expression of LPS-sensitive CD14 and TLR4 receptors (Hamano et al., 2006; Siniavin et al., 2020), which could also be a component of CAP mechanism.

One of the immune cell types involved in sustaining neuroinflammation and innate immunity are mast cells, which abundantly express P2X7 receptors (Kurashima et al., 2012; Wareham and Seward, 2016; Koroleva et al., 2019; Nurkhametova et al., 2019). Mast cells are important players in migraine (Levy, 2009; Kilinc et al., 2017) and proposed recently as essential contributors to Alzheimer’s disease (Kempuraj et al., 2017, 2019). However, there are the limited data on the presence and mechanisms of CAP in mast cells. Although the CAP is often considered as a common phenomenon, it could, however, be a cell-specific mechanism (Savio et al., 2018). Several studies have indicated that nicotine exposure can augment, rather than improve, the pathological process as predicted by CAP. For instance, nicotinic activation of mast cells exaggerates a number of pathological processes including asthma (Yu et al., 2018), pain (Lopes et al., 2016), and atherosclerosis (Wang et al., 2017).

Mast cells, along with P2X7, express also acetylcholine receptors (Sudheer et al., 2006; Mikhailov et al., 2017) suggesting a possible crosstalk between purinergic and cholinergic pathways, in particular, in meningeal tissues densely innervated by cholinergic parasympathetic nerve fibers (Mikhailov et al., 2017).

At molecular level, the direct negative crosstalk between Cys-loop receptors (5-HT3, GABAA, and nicotinic receptors) and certain subtypes of P2X receptors is well established (Sokolova et al., 2001; Boué-Grabot et al., 2003; Limapichat et al., 2014). However, it is unknown whether this type of direct negative crosstalk between nicotinic and the proinflammatory P2X7 receptors occurs, as a hallmark of CAP, in immune cells.

Thus, despite the well-recognized role of P2X7 receptors in induction of inflammasome and IL-1β maturation and release, it remains unclear whether CAP disables the P2X7 receptor function or acts at later steps of the proinflammatory cascade. Here, using the unique ability of P2X7 receptor to open the pathways for large organic molecules such as YO-PRO1 and EtBr, we directly tested whether cholinergic agonists inhibit P2X7 receptor activation in mast cells and macrophages.

## Materials and Methods

### Animals

Experiments were performed on 10- to 12-week male C57BL mice obtained from the Animal Facilities of the University of Eastern Finland (UEF) and approved by the Committee for the Welfare of Laboratory Animals of the UEF and the Provincial Government of Kuopio. All experiments were performed in accordance with the guidelines of the European Community Council (Directives 2010/63/EEC). All efforts were made to minimize the number of animals used and their suffering.

### Isolation of Peritoneal Mast Cells

Peritoneal mast cells were isolated as described previously by Meurer et al. (2016) with slight modifications. In short, animals were anesthetized with inhalation of CO_2_ and sacrificed by cervical dislocation. Using a 10-ml syringe, 3 ml of phosphate-buffered saline (PBS) and 2 ml of air were injected into the peritoneal cavity. Then the animal’s abdomen was carefully shaken using a Pasteur pipette, PBS was collected in the tube and filtered through 100-μm filters (Sysmex CellTrics^®^, Germany). The obtained cell suspension was supplemented with 2% fetal bovine serum (FBS). The lavage procedure was performed using ice-cold solutions, and all following steps were conducted at 4°C to improve mast cell survival and reduce baseline activation level. Collected cell suspension was centrifuged at 300× *g* for 5 min at 4°C. The pellet was resuspended in PBS, and cells were further used for stimulation and staining.

### Identification of Mast Cells

Mast cells were identified using flow cytometry. Freshly isolated peritoneal cells were stained with monoclonal antibodies—anti-mouse FcεRI conjugated with Alexa Fluor^®^ 647 (clone MAR-1, BioLegend, San Diego, CA, USA) and CD117 (c-kit) conjugated with tandem dye APC/Cy7 (clone 2B8, BioLegend, San Diego, CA, USA). Staining protocol was conducted in accordance with the manufacturer’s recommendations. Samples were stained for 15 min on ice in the dark, washed with PBS supplemented with 2% FBS (300 g for 5 min), and resuspended in 300 μl of fresh PBS. Cell viability was determined using 7-AAD (BD Biosciences, San Jose, CA, USA). The data were acquired using CytoFLEX S flow cytometer (Beckman Coulter, USA). Mast cells were identified by double-positive expression of FcεRI and CD117.

### Lipopolysaccharide Priming of Mast Cells

Isolated peritoneal cells were centrifuged at 300× *g* for 5 min, and the pellet was resuspended in RPMI 1640 medium supplemented with 10% FBS. *E. coli* lipopolysaccharide O111:B4 (Sigma–Aldrich, Germany), 1 μg/ml, was added, and the cells were incubated at 37°C for 5 h. Then the cells were washed once and further used for stimulation with ATP and assessment of P2X7 receptor opening using flow cytometry.

### Monocyte-Derived Macrophage Culturing

Monocytes were isolated from the venous blood of healthy volunteers. All participants gave written informed consent prior to the study in accordance with the 2013 Declaration of Helsinki. The study was approved by the local ethics committee of the Pirogov Russian National Research Medical University (Moscow, Russia; protocol #169 from 20.11.2017). Mononuclear cells were isolated by Ficoll-Paque Plus density centrifugation (GE Healthcare, Pittsburgh, PA, USA). Mononuclear cells were resuspended in complete RPMI 1640 medium (supplemented with 10% FBS, 1× GlutaMAX, and 1× penicillin–streptomycin solution; all from RBL Life Technologies, Waltham, MA, USA), plated (1 × 10^6^/ml) in six-well plates, and incubated at 37°C (5% CO_2_). After 90 min, nonadherent cells were removed. To generate monocyte-derived macrophages (MDMs), 100 ng/ml of granulocyte–macrophage colony–stimulating factor (GM-CSF, SCI-store, Russia) was added to the isolated monocytes, and cells were cultured for 6 days.

### Identification of Monocyte-Derived Macrophages

MDMs were detached and analyzed by MACSQuant Analyzer 10 Flow Cytometer (Miltenyi Biotec, Bergish Gladbach, Germany). Forward and side scatter light was used to measure size and granularity of MDMs. For detection of MDM cell surface markers, samples were incubated with monoclonal mouse anti-human antibodies HLA-DR-FITC (clone LN3), CD11b-PE/Cy7 (clone ICRF44), and CD54-Alexa Fluor 488 (clone HCD54) or isotype-matching antibodies (all from Sony Biotechnology, CA, USA) for 30 min on ice. Then samples were washed, resuspended in PBS, and 50,000/sample events were recorded.

### MDM Staining With α-Bungarotoxin

MDMs were plated on poly-L-lysine slides in FluoroBrite DMEM Media and stained with Alexa Fluor-555 α-bungarotoxin (100 nM) for 30 min at 37°C (all from Thermo Fisher Scientific, Waltham, MA, USA). Slides were washed, fixed in buffered 4% paraformaldehyde, coated with a carbonate-buffered glycerin, and examined with an Olympus (Japan) epifluorescence microscope with a CAM-XM10 charge-coupled device (CCD) equipped with appropriate filter sets. Images were analyzed with the ImageJ software (NIH, USA).

### Assessment of ATP-Mediated P2X7 Receptor Large Pore Opening

To assess the activation of P2X7 receptors in peritoneal mast cells, the cells were treated with ATP (Sigma–Aldrich, Germany) alone or in the presence of acetylcholine (Sigma–Aldrich, Germany) or nicotine (RBI, MA, USA). P2X7-gated large pore opening was assessed using the fluorescent dye YO-PRO1 (Thermo Fisher Scientific, USA), known for its ability to penetrate cell membrane through opened P2X7 receptor ion channels (Michel et al., 1999; Browne and North, 2013; Browne et al., 2013; Karasawa et al., 2017). Acetylcholine at a final concentration of 250 μM, nicotine 100 μM, or P2X7 antagonist A839977 5 μM or PBS were added to the samples for 10 min followed by the addition: 1 mM or 5 mM of ATP or PBS. After 10 or 20 min of incubation, YO-PRO1 at a final concentration of 1 μM was added. Additionally, the same protocol was performed in the presence of 50 μM tubocurarine (Sigma Aldrich, Germany). Most of the procedures were performed on ice. In addition, to confirm that low temperature did not affect the results, we performed key experiments at room temperature. Samples were analyzed on a CytoFLEX S flow cytometer (Beckman Coulter, USA); 2,035 ± 81 mast cells were analyzed in each sample. Cell viability within the population of the mast cells was 89.8 ± 1.18%. The data are shown as a percentage of YO-PRO1-positive cells (Karmakar et al., 2016).

In MDMs, P2X7-induced pore formation was assessed as previously described (Constantinescu et al., 2010). Briefly, MDMs resuspended in NaCl medium (145 mM NaCl, 5 mM KCl, 5 mM glucose, 0.1% bovine serum albumin, and 10 mM HEPES, pH 7.5) were incubated with 20 μM EtBr in the presence of ATP for 20 min at 37°C. To assess the effect of cholinergic agents on ATP-induced ethidium uptake, cells were preincubated with 100 μM nicotine or 250 μM acethylcholine for 10 min at 37°C. Incubation was stopped by the addition of an equal volume of ice-cold NaCl solution containing 20 mM MgCl_2_ followed by centrifugation (1,400 rpm for 5 min). Cells were washed once with NaCl solution. Data were acquired using flow cytometry. The EtBr uptake was determined using FlowJo software.

### Whole-Cell Patch Clamp Recordings

To test the presence of nicotinic receptors in MDM cells, the whole-cell patch-clamp (HEKA Elektronic, Germany) recordings from these cells were performed in the extracellular solution (140 mM NaCl, 2 mM CaCl_2_, 2.8 mM KCl, 4 mM MgCl_2_, 20 mM HEPES, and 10 mM glucose; pH 7.4). The microelectrodes (resistance 6–8 MOhm) were pulled from glass capillaries using Narishige PC-10. Internal solution with pH 7.3 contained 140 mM CsCl, 6 mM CaCl_2_, 2 mM MgCl_2_, 2 mM MgATP, 0.4 mM NaGTP, 10 mM HEPES/CsOH, and 20 mM BAPTA/KOH. Cells were clamped at −40 mV and washed in continuous extracellular solution flow containing 10 μM PNU 120596 α7 nAChR-positive modulator. Fast Step (Warner Instrument, USA) application system was set to 5- or 10-s step pulses to deliver the selective α7 agonist PNU 282987 (1 μM in the presence of 10 μM PNU 120596, both from Tocris).

### Statistical Analysis

Flow cytometric data were analyzed using CytExpert Software v.2.3, Kaluza™ software v.1.5a (Beckman Coulter, USA) and FlowJo software 10.0.8 (Three Star Inc., Ashland, OR, USA). Statistical analysis was performed using Origin 8 (Origin labs, MS, USA) and GraphPad Prism 4 (GraphPad Software, La Jolla, CA, USA). In mouse study, *n* represents the number of animals within one group of treatment. Data were analyzed using one-way ANOVA, followed by Bonferroni’s multiple comparisons test. A *p*-value less than 0.05 was considered significant. The data are presented as mean ± SEM.

The raw data supporting the conclusions of this manuscript will be made available by the authors, without undue reservation, to any qualified researcher.

## Results

### Testing Action of Cholinergic Agonists on ATP-Induced YO-PRO1 Uptake in Mast Cells

**Figure 1:**
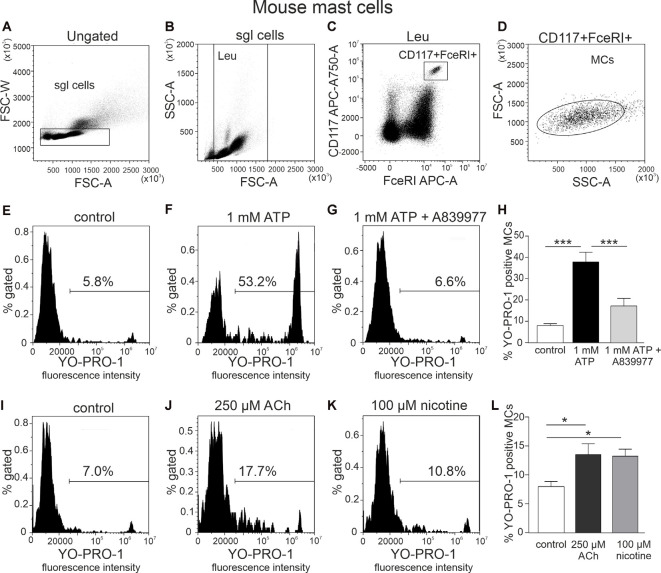
Flow cytometric gating strategy for identification of mast cells and testing action of ATP, acethylcholine, and nicotine on the YO-PRO1 uptake by peritoneal mast cells. **(A)** Single cells gating based on FSC-A vs. FSC-W (the “sgl cells” region is set to discriminate cell doublets). **(B)** Light scatter dot plot for cells based on forward scatter (FSC-A) and side scatter (SSC-A) [leucocyte (Leu) region is set to discriminate debris]. **(C)** Cell subpopulations based on Alexa Fluor 647-FcεRI and APC-Cy7-CD117 (the region is set to identify double-positive cells). **(D)** Backgating of double-positive cells onto the light scatter plot FSC-A vs. SSC-A. This shows relative size and granularity of mast cells compared to those in **(B)**. Cells from the region of “mast cells” were further used to assess the effects of ATP, acetylcholine, and nicotine. **(E–G,I–K)** Representative histograms (fluorescence intensity vs. percent of gated mast cells) of YO-PRO1 uptake by mast cells. **(E,I)** Negative control [treated with phosphate-buffered saline (PBS)], mouse peritoneal mast cells incubated with 1 μM of YO-PRO. **(F)** Mast cells incubated with 1 mM of ATP for 20 min. **(G)** Mast cells preincubated with P2X7 antagonist A839977 (5 μM), followed by stimulation with 1 mM ATP. **(H)** Histograms showing percent of YO-PRO1-positive cells in control (white, *n* = 12), after stimulation with 1 mM ATP (black, *n* = 12) and in the presence of the P2X7 antagonist A839977 (5 μM; gray, *n* = 9). **(J)** Mast cells incubated with 250 μM acetylcholine. **(K)** Mast cells incubated with 100 μM nicotine. **(L)** Histograms showing percent of YO-PRO1-positive cells in control (white, *n* = 12), after stimulation with 250 μM acetylcholine (black, *n* = 5) and 100 μM nicotine (gray, *n* = 5). Mean ± SEM, *p < 0.05, ***p < 0.0001 (one-way ANOVA, followed by Bonferroni’s multiple comparison test).

Mast cells were identified as population double positive for both FcεRI and CD117, which were further evaluated for their ability to show P2X7 receptor activation. The details of the gating strategy are shown in Figures 1A–D.

YO-PRO1 dye was used to detect the opening of P2X7 receptor channels, specifically, the large pore (Surprenant et al., 1996; Pelegrin and Surprenant, 2006) associated with inflammasome activation. Relatively high concentrations of ATP were used since P2X7 receptors have a low affinity to this purinergic agonist (North, 2002). Our data show that stimulation of freshly isolated mast cells with 1 mM ATP caused a significant increase in percentage of YO-PRO1-positive cells (8.0 ± 0.9% in control, up to 37.8 ± 4.6% after stimulation with ATP, *n* = 12, *p* < 0.001; Figures 1E,F,H). This increase was completely inhibited by the specific P2X7 receptor antagonist 5 μM A839977 (*n* = 9, *p* < 0.001; Figures 1G,H; Honore et al., 2009), confirming that YO-PRO1 uptake was mediated *via* P2X7-gated ion channels. Surprisingly, incubation with 250 μM acetylcholine induced a small, but significant, increase in the percentage of YO-PRO1-positive mast cells (13.5 ± 1.9%, *n* = 5, *p* < 0.05; Figures 1I,J,L). A similar effect was observed also with 100 μM nicotine, which increased the percentage of YO-PRO1-positive cells up to 13.2 ± 1.2% (*n* = 5, *p* < 0.05; Figures 1K,L). To assess whether this small effect is associated with the opening of the large pore of P2X7 receptor, we repeated this test after preincubation of mast cells with the P2X7 antagonist A839977. We found that in the presence of A839977, the percentage of YO-PRO1-positive cells, after the application of Ach and nicotine, slightly decreased (Supplementary Figure 1); however, this effect was not statistically significant (*p* > 0.05 for both cholinergic ligands).

**Figure 2:**
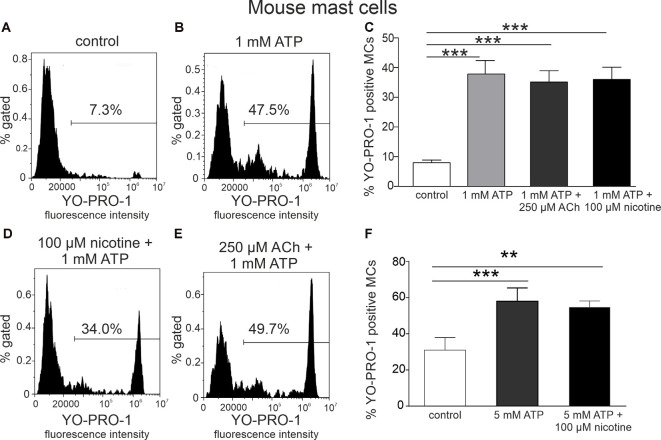
Acethylcholine and nicotine do not affect ATP-stimulated YO-PRO1 uptake. **(A,B,D,E)** Representative histograms (fluorescence intensity vs. percent of gated mast cells) of YO-PRO1 uptake by mast cells. **(A)** Negative control (cells treated with PBS), mouse peritoneal mast cells incubated with 1 μM of YO-PRO1. **(B)** Mast cells incubated with 1 mM of ATP. **(D)** Mast cells preincubated with 100 μM nicotine followed by stimulation with 1 mM ATP. **(E)** Mast cells preincubated with 250 μM acetylcholine followed by stimulation with 1 mM ATP. **(C)** Histograms showing percent of YO-PRO1-positive cells in control (white, *n* = 12), after stimulation with 1 mM ATP (light gray, *n* = 12) alone, and in the presence of 250 μM acetylcholine (dark gray, *n* = 12) or 100 μM nicotine (black, *n* = 12). **(F)** Histograms showing percent of YO-PRO1-positive cells in control (white, *n* = 3), after stimulation with 5 mM ATP (gray, *n* = 3) alone, and in the presence of 100 μM nicotine (black, *n* = 12). Mean ± SEM, **p < 0.01, ***p < 0.001 vs. control (one-way ANOVA, followed by Bonfferoni’s multiple comparison test).

As ATP produced a robust opening of P2X7 ion channel to YO-PRO1, we next tested the key question, whether incubation of these immune cells with the cholinergic agent inhibits this large channel opening indicative of anti-inflammatory response. However, acetylcholine (250 μM) and nicotine (100 μM) did not decrease ATP-triggered activation of mast cells (Figures 2C–F), compared to stimulation with ATP alone (*p* > 0.05; Figures 2A–C). A similar lack of depression of YO-PRO1 uptake by nicotine was obtained when performing experiments at room temperature (Supplementary Figure 2), in the presence of the nicotinic antagonist d-tubocurarine (Supplementary Figure 3) or when we tested this effect at a shorter time interval (Supplementary Figure 4).

To address whether the priming of the NLRP3 inflammasome with LPS (Gicquel et al., 2014; Parzych et al., 2017) is critical for the modulation of P2X7 receptors with nicotine, we tested the action of this agonist on ATP-stimulated YO-PRO1 uptake in mast cells. However, again, no depression of the YO-PRO1 response by nicotine was observed even in these proinflammatory conditions (Supplementary Figure 5).

### Nicotinic Agonists Generate Membrane Currents but Do Not Suppress Large Pore Opening of P2X7 Receptor in Human Macrophages

**Figure 3:**
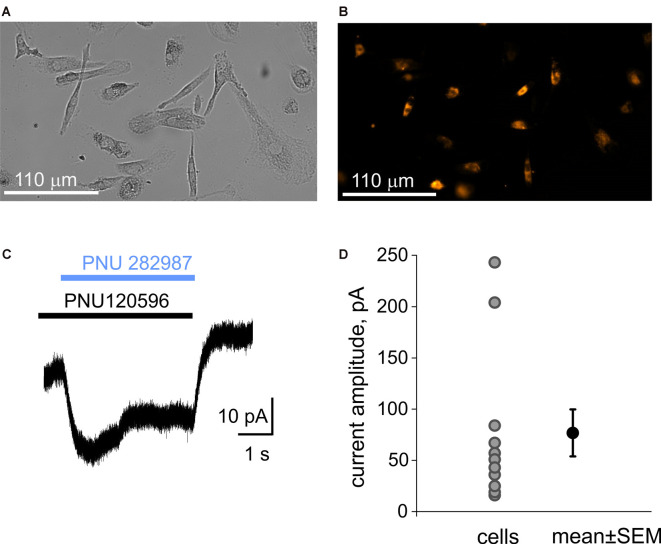
Immunolabeling of α7 receptors and patch-clamp recording of α7 receptors mediated currents from macrophages (MDM cells). **(A)** A transparent light image of monocyte-derived macrophage (MDM) cells. **(B)** Detection of α7 receptors with fluorescently labeled α-bungarotoxin. **(C)** Representative example of the membrane current activated by 1 μM PNU 282987 together with 10 μM PNU 120596 on MDM cells. Black and blue rectangles represent application of PNU120596 and PNU282987, respectively. **(D)** Plot showing amplitudes of responses in individual MDM cells (77 ± 23 pA, *n* = 11).

We first examined the expression of α7 nicotinic receptors in human macrophages (MDM cells), a common model to study CAP. The labeling of MDM cells with the fluorescently labeled α7 antagonist α-bungarotoxin (α-BTX) revealed the abundance of positive cells (Figures 3A,B). Likewise, patch-clamp recordings demonstrated the functional expression of α7 receptors on the plasma membrane of the MDMs. Figure 3C shows the current trace recorded from MDMs under co-application of the selective α7 receptor agonist PNU 282987 (1 μM) with the selective α7 receptor-positive allosteric modulator PNU 120596 (10 μM). This combination was used to overcome the strong desensitization of α7 receptors (Siniavin et al., 2020). Eleven tested MDMs have shown ion currents (77 ± 23 pA, *n* = 11) upon application of PNU 282987 plus PNU 120596 (Figure 3D) suggesting a functional expression of α7 receptors.

**Figure 4:**
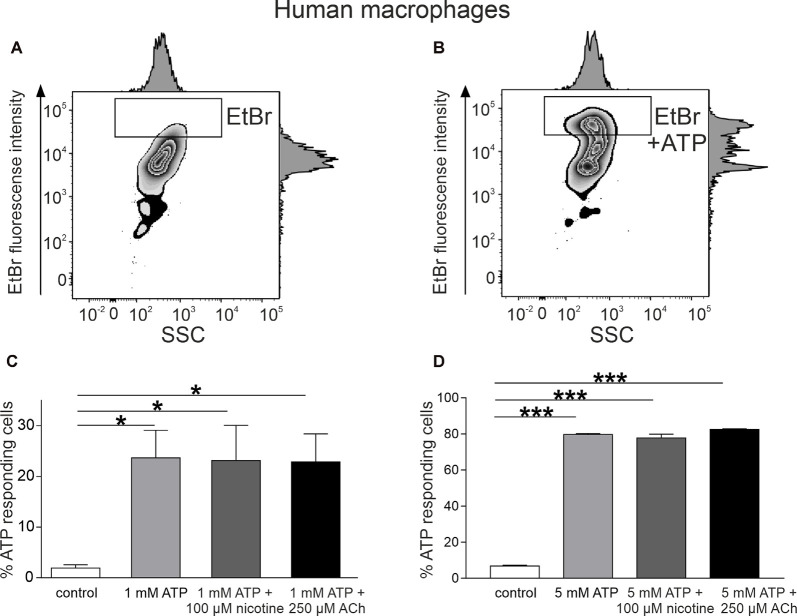
ATP-induced ethidium bromide (EtBr) uptake into monocyte-derived macrophages (MDMs). **(A)** Representative histogram of EtBr uptake in control. **(B)** Representative histogram of EtBr uptake in the presence of 1 mM ATP at 37°C for 10 min. **(C)** Histograms showing percent of EtBr-positive cells in control (white, *n* = 5), in the presence of ATP 1 mM (light gray, *n* = 5) alone, and after preincubation with 100 μM nicotine (dark gray, *n* = 5) or 250 μM acetylcholine (black, *n* = 5). **(D)** Histograms showing percent of EtBr-positive cells in control (white, *n* = 5), in the presence of ATP 5 mM (light gray, *n* = 3) alone, and after preincubation with 100 μM nicotine (dark gray, *n* = 3) or 250 μM acetylcholine (black, *n* = 3). Mean ± SEM, *p < 0.05, ***p < 0.0001 (one-way ANOVA, followed by Tukey’s *post hoc* test).

For testing whether cholinergic stimulation can diminish the large pore opening after ATP application in MDM cells, we used the EtBr uptake assay (Pelegrin and Surprenant, 2006; Schachter et al., 2008; Lemaire et al., 2011; gating strategy is shown in Supplementary Figure 6). We found that 10 min of incubation with ATP induced a significant uptake of this dye into MDMs (Figures 4A–D). Thus, in the presence of 1 mM ATP, proportion of responding cells increased from 1.9 ± 0.6% to 23.6 ± 5.4% (*n* = 5, *p* < 0.05). After incubation with 5 mM ATP, the proportion of ethidium-labeled cells was 87.3 ± 0.5% vs. 7.0 ± 0.8% in the control (*n* = 3). Nevertheless, like with mast cells, neither acetylcholine (250 μM) nor nicotine (100 μM) reduced the ATP-induced ethidium uptake (Figures 4C,D).

Taken together, our data show the lack of the inhibitory action of nicotinic agents on ATP-driven activation of P2X7 receptors leading to opening of a large pore, either in mast cells or in macrophages, suggesting that CAP likely acts downstream of active P2X7 receptors.

## Discussion

Here, we, for the first time, investigated the potential inhibitory role of the cholinergic agents, acetylcholine and nicotine, on ATP-triggered activation of the large pore of P2X7 receptor in mast cells and human macrophages. We demonstrate that in both cell types, ATP opens the large pore of P2X7 receptors, typically associated with activation of inflammasome, and this effect was blocked by the specific P2X7 antagonist. However, in both types of immune cells, including macrophages, in which we proved the function of α7 nicotinic receptors, acetylcholine and nicotine failed to reduce the P2X7 receptor large channel opening. Notably, acetylcholine and nicotine slightly, but significantly, increased YO-PRO1 uptake in mast cells.

Both purinergic and cholinergic signaling are currently recognized as important contributors to the inflammatory processes. However, accumulating evidence suggests that they have opposite roles: purinergic cascades promote, whereas the cholinergic pathway inhibits, the induction of inflammation. In the current study, by using mast cells and human macrophages as a model of immune cell, we addressed whether these receptor pathways intersect at the initial steps of inflammatory activation.

The concept of “cholinergic anti-inflammatory pathway” (CAP), initially introduced by Borovikova et al. (2000), has recently gained attention from the scientific community. According to this view, activation of CAP *via* α7 nicotinic receptor leads to reduction of inflammatory response and subsequent cytokines release (Wang et al., 2003). These α7 receptors were discussed as mediators of CAP in Alzheimer’s disease (reviewed in Benfante et al., 2019). Likewise, it has been proposed that α7 receptors mediate the anti-inflammatory effect in chronic migraine (Liu et al., 2018).

Ligand-gated α7 receptors were found in various immune cells, including peripheral macrophages (Wang et al., 2003; Ke et al., 2017; Siniavin et al., 2020) and mast cells (Sudheer et al., 2006). Macrophages are very common cell types to study the mechanisms of CAP (Báez-Pagán et al., 2015). In contrast, CAP phenomena are little studied in mast cells. However, mast cells are known as the powerful mediators of inflammation acting *via* different mechanisms (Galli and Tsai, 2012; Wernersson and Pejler, 2014). In addition to acute degranulation and release of classical mediators such as serotonin and histamine (Giniatullin et al., 2019; Koroleva et al., 2019), activation of mast cells leads to production of multiple cytokines, including IL-1β (Theoharides et al., 2012; Cardamone et al., 2016). Consistent with the mechanism of CAP, earlier studies have demonstrated that nicotine inhibits degranulation of mast cells (Kageyama-Yahara et al., 2008), as well as of leukotriene and cytokine production (Mishra et al., 2010). Furthermore, the stimulation of α7 receptors on mast cells diminishes the production of proinflammatory cytokine TNF-α (Guzmán-Mejía et al., 2018). On the other hand, nicotinic activation can exaggerate some mast cell-associated pathological processes such as asthma (Yu et al., 2018).

Purinergic regulation of immune reactions is largely mediated by P2X7 receptors, which are involved in the proinflammatory responses *via* induction of inflammasome and IL-1β production (Grahames et al., 1999; Pelegrin and Surprenant, 2006; Gross et al., 2011; Rathinam et al., 2012; Franceschini et al., 2015; Cekic and Linden, 2016; Karmakar et al., 2016). The activation of P2X7 receptors and associated K^+^ efflux is a key event in the activation of inflammasome (Muñoz-Planillo et al., 2013). We and others have shown that mast cells express functional proinflammatory P2X7 receptors (Kurashima et al., 2012; Wareham and Seward, 2016; Nurkhametova et al., 2019; Shen et al., 2020, current study). P2X7 receptor signaling was proposed to be enhanced in sensitized (primed) mast cells (Di Virgilio et al., 2018). However, in our study, the priming of inflammasome with LPS (Gicquel et al., 2014; Parzych et al., 2017) did not promote the action of nicotine on large pore formation in mast cells.

Here, we selected the large pore opening as an indicator of the ability of P2X7 receptors to activate inflammasome (Ferrari et al., 2006; Franceschini et al., 2015; Di Virgilio et al., 2017; Wang et al., 2020). Our findings revealed a lack of direct inhibitory effect of cholinergic agents on the early steps of ATP-mediated activation leading to large pore formation. This result was demonstrated both in mast cells and macrophages and in all tested conditions. Besides, our results revealed a small, but significant, effect of both acethylcholine and nicotine on the uptake of YO-PRO1 in mast cells, which was not observed on the MDM cells. One possibility was that this small mast cell-specific effect of acetylcholine might be due to massive degranulation of mast cells expressing muscarinic receptors (Mikhailov et al., 2017) and release of endogenous ATP (Suleimanova et al., 2020). However, our testing of the action of ACh and nicotine in the presence of the specific P2X7 receptor antagonist did not reveal the action of these cholinergic agents *via* ATP-dependent mechanism. Nicotine does not degranulate mast cells (Mikhailov et al., 2017), although evidence suggest the expression of nicotinic receptors in these cells (Sudheer et al., 2006; Guzmán-Mejía et al., 2018). Moreover, this action of nicotine may be due to its known action on TRPA1 receptors (Kichko et al., 2013) expressed in mast cells (Oh et al., 2013). Interestingly, the α7 agonist might be silent (Papke and Lindstrom, 2020), whereas the classical nicotinic antagonist tubocurarine has been shown to activate peritoneal mast cells (Tsvilovskyy et al., 2020) suggesting a noncanonical nicotinic modulation in immune cells.

As a classical model to investigate the possible effects of cholinergic agents on the activation of P2X7 receptors, we used human macrophages where we first confirmed the functional expression of α7 receptors. With macrophages, our main finding on the inability of cholinergic agonists to diminish the activation of the large pore by ATP is similar to the result demonstrated on mast cells.

In conclusion, our data show that ATP induces opening of the P2X7 ion channels permeable to large organic molecules in mouse peritoneal mast cells and human macrophages; however, acetylcholine and nicotine fail to alter this event commonly leading to activation of inflammasome. These data further develop the promising concept of CAP and can help in the search of the novel draggable targets to combat the excessive inflammatory reactions in various immune and neurological disorders.

## Data Availability Statement

The raw data supporting the conclusions of this article will be made available by the authors, without undue reservation.

## Ethics Statement

The animal study was reviewed and approved by Animal Facilities of the University of Eastern Finland (UEF) and approved by the Committee for the Welfare of Laboratory Animals of the University of Eastern Finland and the Provincial Government of Kuopio. Human samples were obtained with written informed consent prior to the study in accordance with the 2013 Declaration of Helsinki. The study was approved by the local Ethics Committee of the Pirogov Russian National Research Medical University (Moscow, Russia; protocol #169 from 20.11.2017).

## Author Contributions

DN and AS contributed to the data collection, analysis, interpretation, and manuscript writing. IK contributed to the data analysis and interpretation. RGiniatullina, DK, and MS contributed to the data collection and analysis. RGiniatullin, VT, and TM contributed to the study design and supervision, manuscript writing, and final editing. All authors contributed to the article and approved the submitted version.

## Conflict of Interest

The authors declare that the research was conducted in the absence of any commercial or financial relationships that could be construed as a potential conflict of interest.
