# Supplementary material for: Does Cholinergic Stimulation Affect the P2X7 Receptor-Mediated Dye Uptake in Mast Cells and Macrophages?
Source: Front Cell Neurosci. 2020 Oct 28;14:548376. doi: 10.3389/fncel.2020.548376 (PMC7673375; doi:10.3389/fncel.2020.548376)
Supplement: Supplementary file 1 [file Data_Sheet_1.PDF]

Supplementary Material

Supplementary Figures

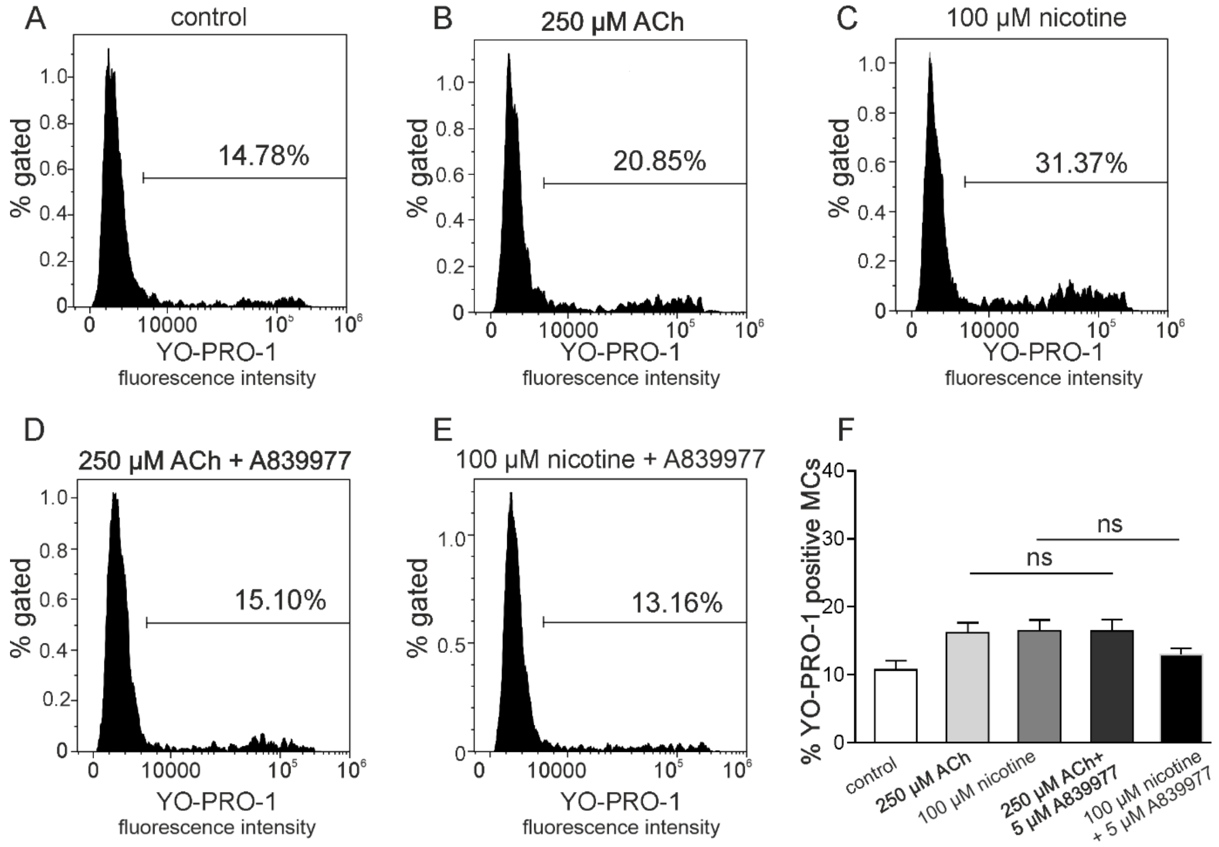

Supplementary Figure 1. YO-PRO1 uptake by mast cells induced by cholinergic agents in the presence of the specific P2X7 antagonist A839977. (A-E) Representative histograms (fluorescence intensity vs. percent of gated mast cells) of YO-PRO1 uptake by mast cells. (A) Control mouse mast cells incubated with 1 µM of YO-PRO1. (B) Mast cells incubated with 250 µM Ach. (C) Mast cells incubated with 100 µM nicotine (D) Mast cells pre-incubated with 5 µM A839977, followed by stimulation with 250 µM Ach. (D) Mast cells pre-incubated with 5 µM A839977, followed by stimulation with 100 µM nicotine. (E) Histograms showing a percent of YO-PRO1 positive cells in control (n=17), after incubation with 250 µM ACh (n=10), 100 µM nicotine (n=10), 250 µM ACh in the presence of 5 µM A839977 (n = 5, p > 0.05 vs ACh) or 100 µM nicotine in the presence of 5 µM A839977 (n = 4, p >0.05 vs nicotine). Mean ± SEM, (one-way ANOVA, followed by Bonferoni's multiple comparison test).

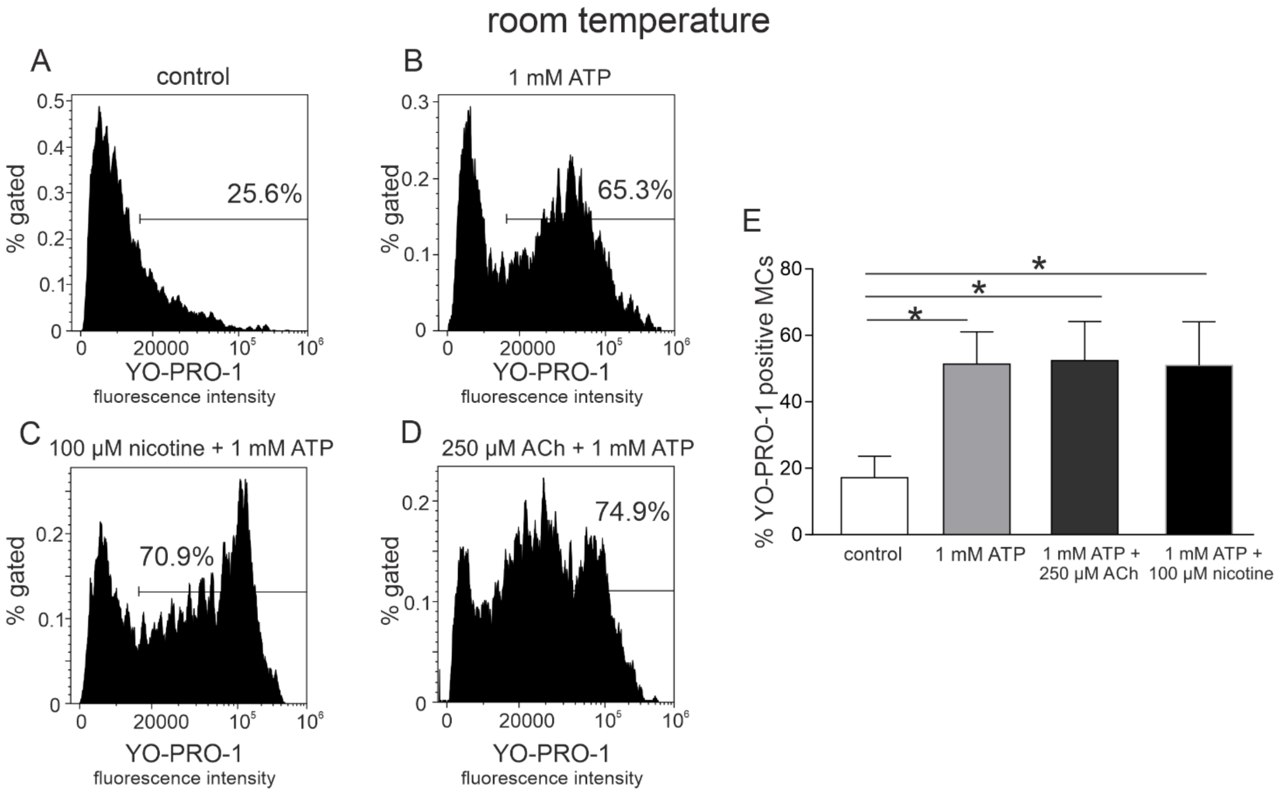

Supplementary Figure 2. Cholinergic agents do not affect ATP-induced YO-PRO1 uptake by peritoneal mast cells (room temperature). (A-C) Representative histograms (fluorescence intensity vs. percent of gated mast cells) of YO-PRO1 uptake by mast cells. (A) Negative control (treated with PBS), mouse peritoneal mast cells incubated with 1 µM of YO-PRO. (B) Mast cells incubated with 1 mM of ATP for 20 minutes. (C) Mast cells pre-incubated with 100 µM nicotine followed by stimulation with 1 mM ATP. (D) Mast cells pre-incubated with 250 µM acethylcholine followed by stimulation with 1 mM ATP. (E) Histograms showing percent of YO-PRO1 positive cells in control (white, 17.2 ± 3.7%, n = 3), after stimulation with 1 mM ATP (light grey, 51.4 ± 5.6%, n = 3) alone, and in the presence of 250 µM acetylcholine (dark grey, 52.4 ± 6.8%, n = 3) or 100 µM nicotine (black, 51.0 ± 7.6%, n = 12). Mean ± SEM, *$p < 0.05$ vs control (one-way ANOVA, followed by Bonfferoni's multiple comparison test).

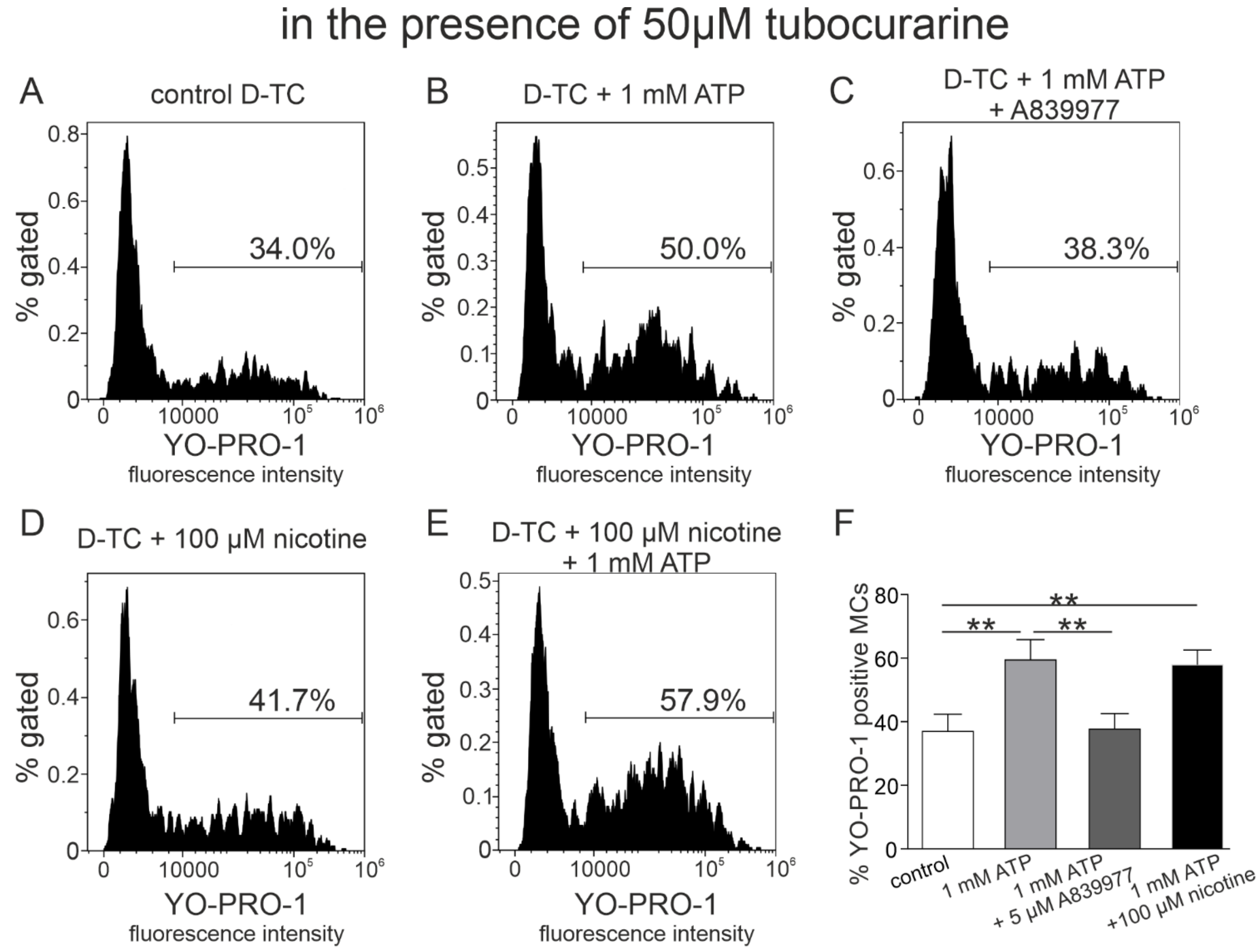

Supplementary Figure 3. YO-PRO1 uptake by mast cells in the presence of 50 μM D-tubocuratine (D-TC). (A-E) Representative histograms (fluorescence intensity vs. percent of gated mast cells) of YO-PRO1 uptake by mast cells. All samples are pre-incubated with 50 μM D-tubocuratine. (A) Negative control (treated with PBS), mouse peritoneal mast cells incubated with 1 μM of YO-PRO. (B) Mast cells incubated with 1 mM of ATP for 20 minutes. (C) Mast cells pre-incubated with 5 μM A839977, followed by stimulation with 1 mM ATP. (D) Mast cells incubated with 100 μM nicotine. (D) Mast cells pre-incubated with 100 μM nicotine followed by stimulation with 1 mM ATP. (E) Histograms showing percent of YO-PRO1 positive cells in control (white, 36.9 ± 3.1%, n = 3), after stimulation with 1 mM ATP (light grey, 59.5 ± 3.7%, n = 3) alone, and in the presence of 5 μM A839977 (dark grey, 37.7 ± 2.8%, n = 3) or 100 μM nicotine (black, 57.9 ± 2.7%, n = 3). Mean ± SEM , **p < 0.01 (one-way ANOVA, followed by Bonfferoni's multiple comparison test).

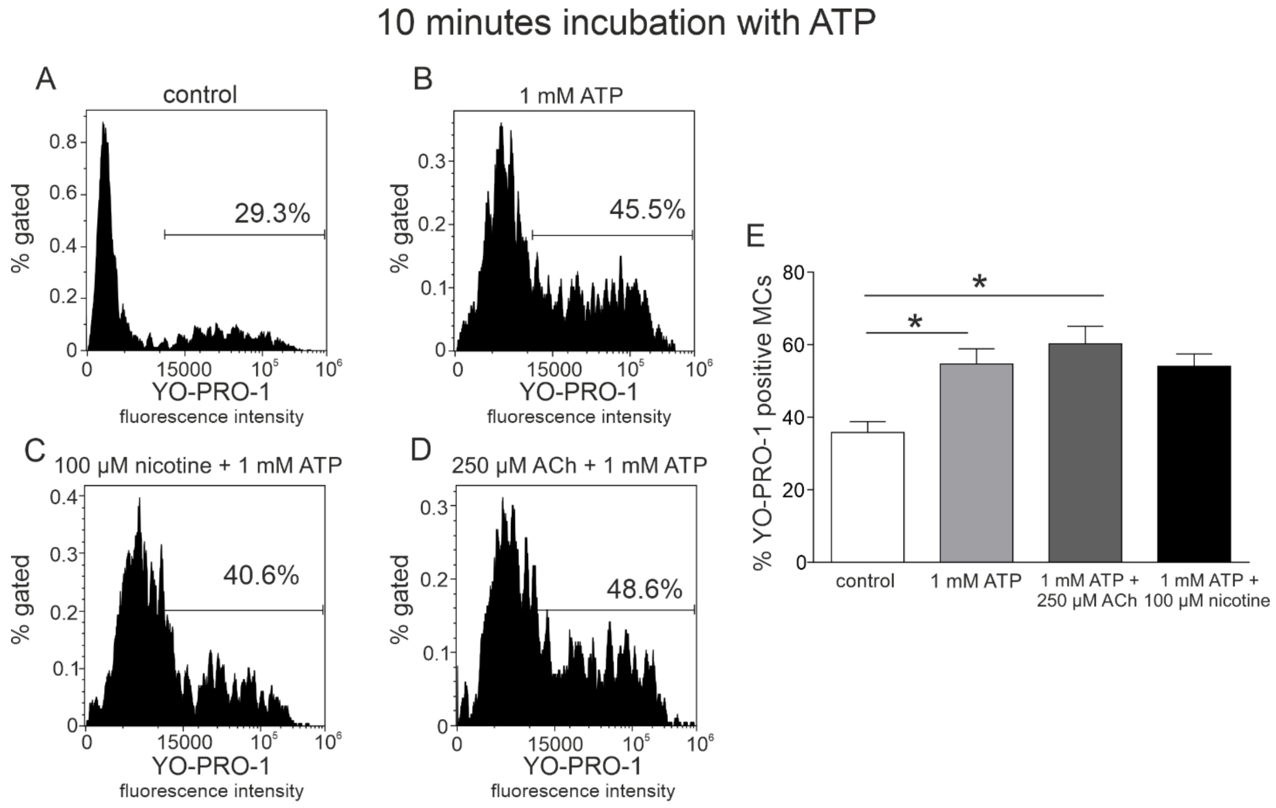

Supplementary Figure 4. YO-PRO1 uptake by mast cells after 10 minutes incubation with ATP (A-E) Representative histograms (fluorescence intensity vs. percent of gated mast cells) of YO-PRO1 uptake by mast cells. (A) Negative control (treated with PBS), mouse peritoneal mast cells incubated with 1 μM of YO-PRO. (B) Mast cells incubated with 1 mM of ATP for 10 minutes. (C) Mast cells pre-incubated with 100 μM nicotine followed by stimulation with 1 mM ATP. (D) Mast cells pre-incubated with 250 μM acethylcholine followed by stimulation with 1 mM ATP. (E) Histograms showing percent of YO-PRO1 positive cells in control (white, 35.8 ± 2.9%, n = 3), after stimulation with 1 mM ATP (light grey, 54.7 ± 4.2%, n = 3) alone, and in the presence of 250 μM acethylcholine (dark grey, 60.3 ± 4.8%, n = 3) or 100 μM nicotine (black, 54.1 ± 3.4%, n = 3). Mean ± SEM ,*p < 0.05 (one-way ANOVA, followed by Bonfferoni's multiple comparison test).

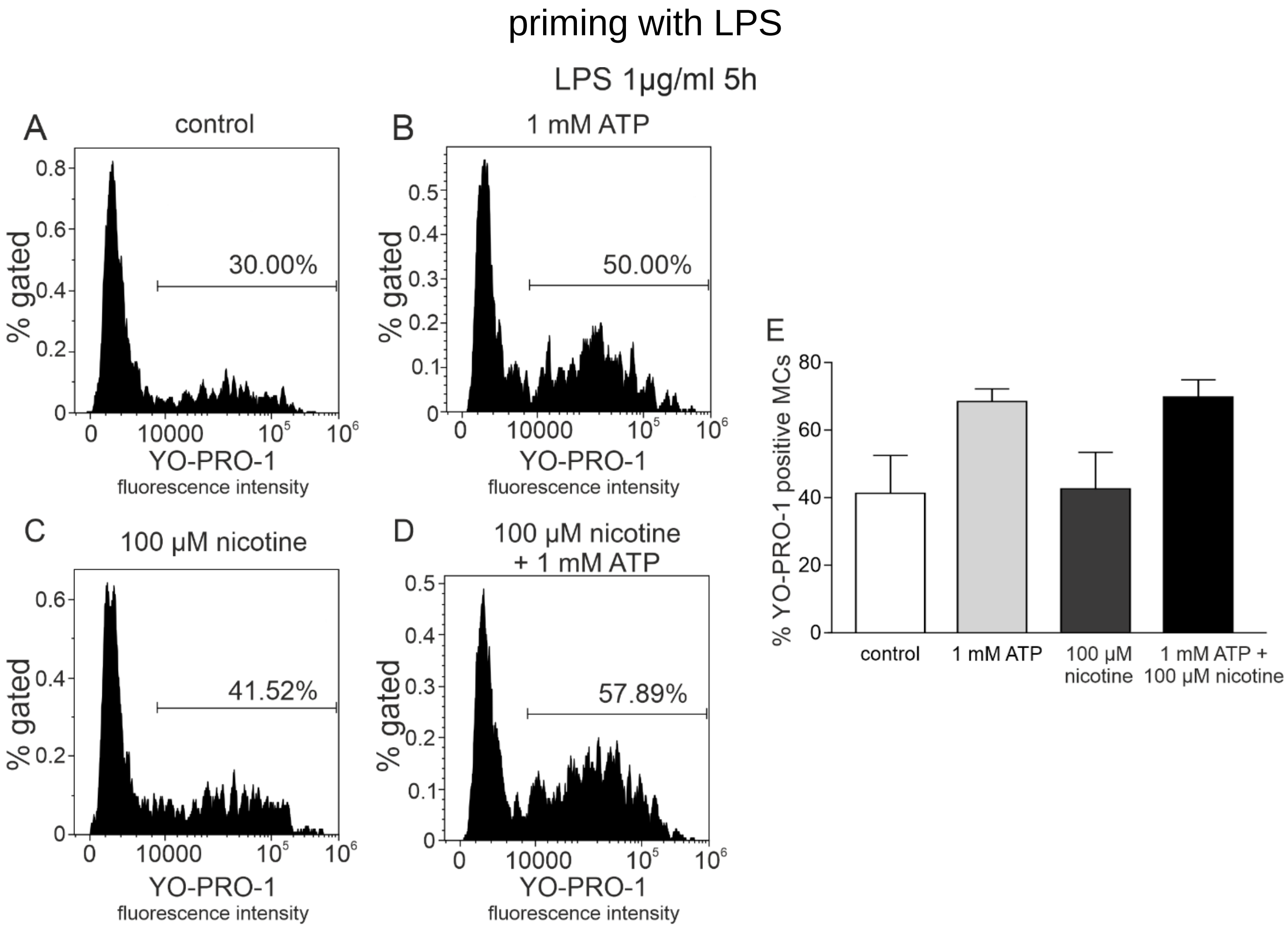

Supplementary Figure 5. YO-PRO1 uptake by mast cells after incubation with LPS 1 µg/ml for 5 hours. **(A-D)** Representative histograms (fluorescence intensity vs. percent of gated mast cells) of YO-PRO1 uptake by mast cells. (A) Negative control (treated with PBS), mouse peritoneal mast cells incubated with 1 µM of YO-PRO. (B) Mast cells incubated with 1 mM of ATP for 20 minutes. (C) Mast cells incubated with 100 µM nicotine (D) Mast cells pre-incubated with 100 µM nicotine followed by stimulation with 1 mM ATP. (E) Histograms showing percent of YO-PRO1 positive cells in control (white, 41.3 ± 11.2%, n = 3), after stimulation with 1 mM ATP (light grey, 68.5 ± 3.8%, n = 3), incubation with 100 µM nicotine (dark grey, 42.4 ± 11.1%, n = 3), and after stimulation with 1 mM ATP in the presence of 100 µM nicotine (black, 69.7 ± 5.2%, n = 3). Mean ± SEM, $p > 0.05$ (one-way ANOVA, followed by Bonfferoni's multiple comparison test).

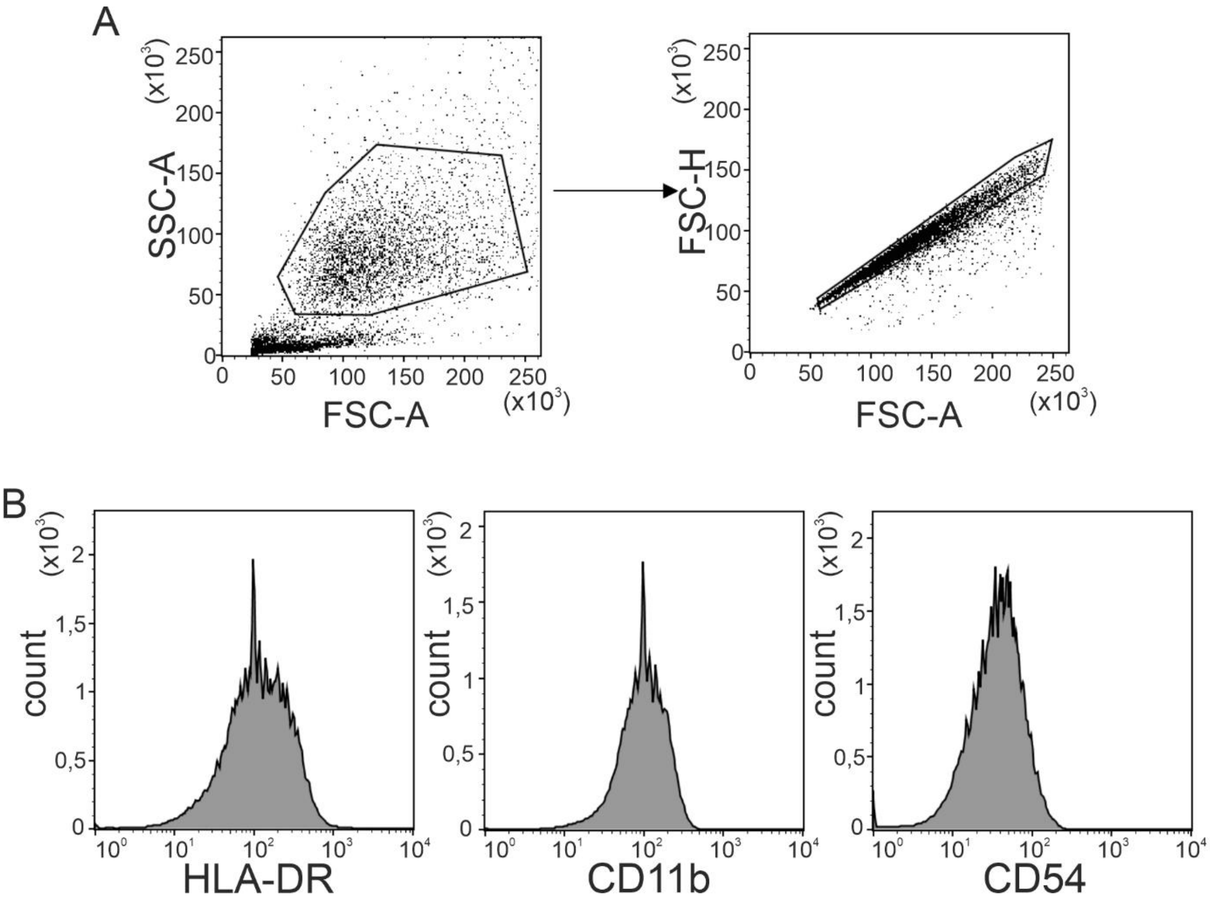

Supplementary Figure 6. MDMs gating strategy for flow cytometry analysis. (A) In this sample gating, cells were first gated for macrophages (SSC-A vs. FSC-A) and singlets (FSC-H vs. FSC-A). (B) The macrophages gate is further analyzed for their expression of HLA-DR, CD11b and CD54.
